# Report on a case of giant retroperitoneal perirenal liposarcoma with kidney preservation

**DOI:** 10.1093/jscr/rjag319

**Published:** 2026-04-28

**Authors:** Lujie Shi, Ke Liao, Jun Zhou

**Affiliations:** Department of General Surgery, Xindu District People's Hospital of Chengdu, Sichuan, China; Precision Medicine Research Center, West China Hospital, Sichuan University, Chengdu, China; Department of General Surgery, Xindu District People's Hospital of Chengdu, Sichuan, China

**Keywords:** retroperitoneal liposarcoma, well-differentiated, perirenal liposarcoma

## Abstract

Retroperitoneal liposarcoma is an uncommon malignant tumor and a subtype of soft tissue sarcoma. Early diagnosis is particularly challenging due to the tumour’s location in the retroperitoneal space, which provides ample room for expansion. Symptoms typically manifest only when the tumor reaches a substantial size or invades adjacent organs. We report a case of a 61-year-old woman who underwent surgical resection for a well-differentiated liposarcoma. Computed tomography scan revealed that the mass invaded the left perirenal fascia and displaced the descending colon, pancreas, and duodenum. Complete resection of tumour masses was performed, and we opted to preserve the kidney due to no visible renal parenchymal tumour invasion. The management of retroperitoneal perirenal liposarcoma typically entails the implementation of an R0 resection. If a tumor extends into an adjacent tissue, one must consider the benefits of achieving clear surgical margins versus the risks of medical issues and reduced quality of life.

## Introduction

Liposarcoma is the most common variant of soft tissue sarcoma. Retroperitoneal liposarcoma (RLPS) is a relatively rare malignant tumor that frequently recurs locally and is difficult to treat, with a poorer prognosis compared to liposarcomas found in the limbs [1]. RLPS represents a distinct subtype of soft tissue sarcoma and accounts for ~10%–15% of soft tissue sarcomas. Notably, approximately one-third of these retroperitoneal liposarcomas originate from perirenal adipose tissue, subsequently developing into perirenal liposarcoma [2, 3]. At present, the American Cancer Society has identified certain risk factors for liposarcoma, including radiation exposure, genetics, lymphatic system damage, and exposure to toxic chemicals [4]. However, there is still no clear cause for liposarcoma. In the retroperitoneal space, which is relatively capacious, liposarcomas often remain asymptomatic in their early stages. By the time of detection, these tumors typically reach a substantial volume and may encroach upon adjacent organs, including the colon, kidneys, and vena cava. Achieving wide excision with a secure surgical margin proves to be a formidable challenge. Furthermore, the local recurrence rate of these tumors following resection is notably high. Due to the large size of the tumor, it often displaces or invades surrounding organs and blood vessels, making the surgical operation quite challenging. In such cases, it is often necessary to jointly remove the surrounding organs [5]. However, some experts hold the view that nephrectomy should only be performed when complete removal of the tumor is necessary for visual inspection purposes [6]. Currently, there is still some controversy regarding the optimal scope of surgical resection.

## Presentation of case

**Figure 1:**
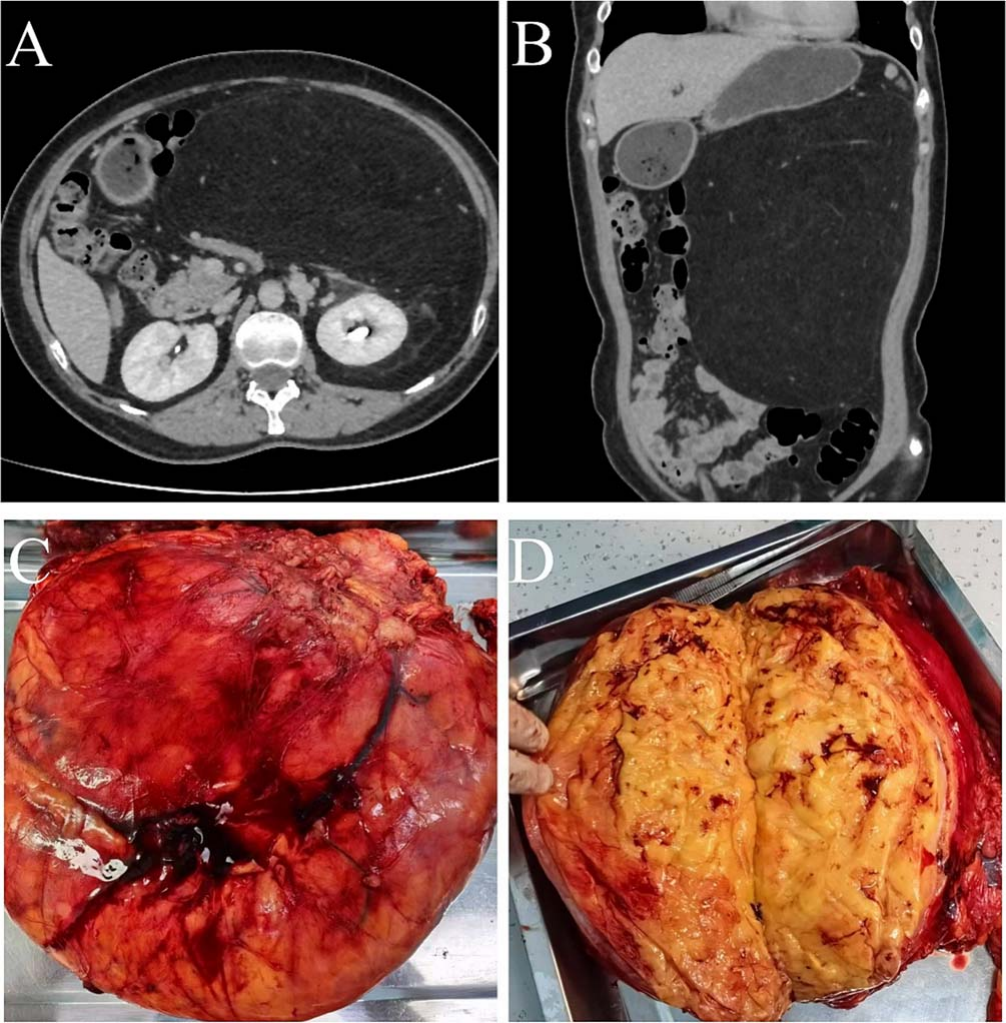
(A and B) CT detected a fatty density lesion in the retroperitoneal space. (C and D) The mass measured 32 × 24 × 7 cm, and had with heterogeneous yellowish mass appearance.

**Figure 2:**
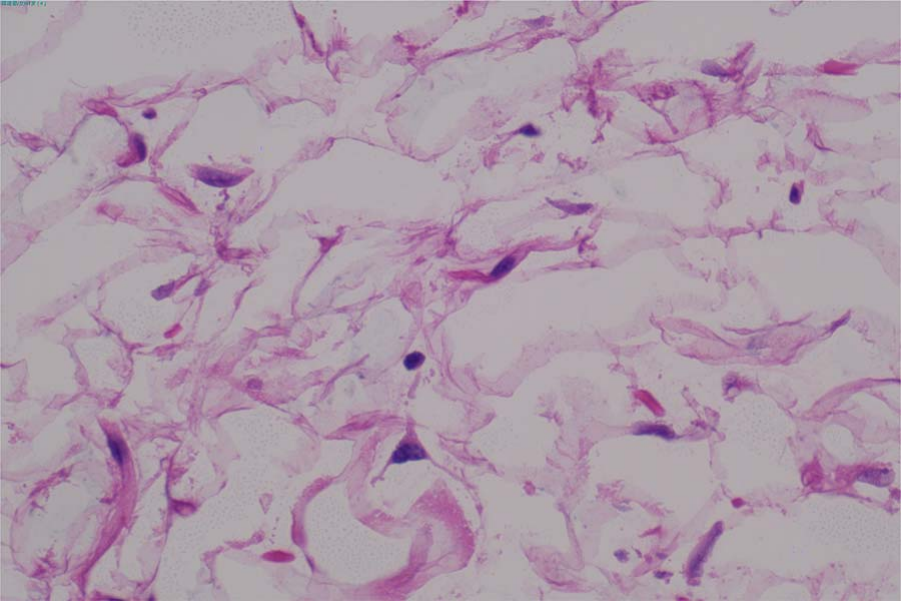
Postoperative histopathologic examination reveals well-differentiated liposarcoma.

**Figure 3:**
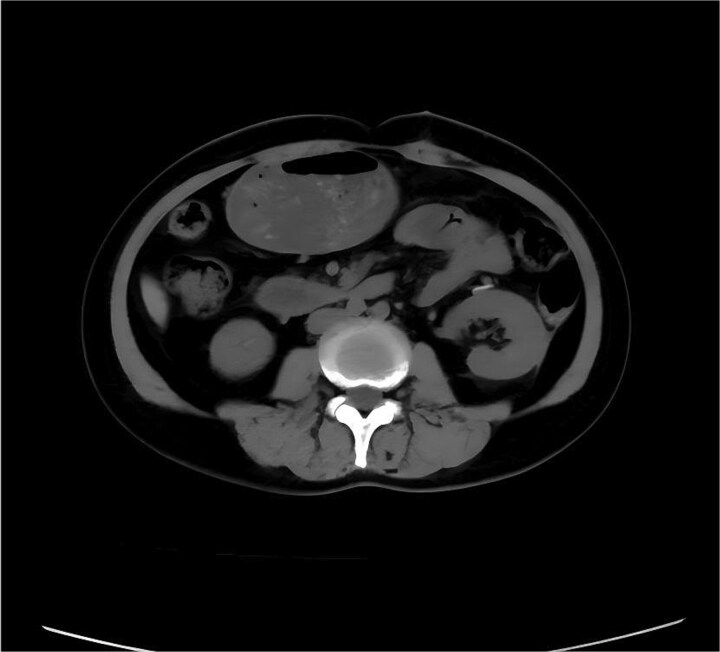
The CT scan showed no recurrence after 12 months following surgery.

A 61-year-old female patient was hospitalized due to abdominal pain for a day and recurrently difficult bowel movements for over 5 years. Laboratory examinations of complete blood counts, urine tests, and tumor markers were otherwise normal. CT enhanced scan showed a large mass of predominantly fat density in the left retroperitoneum and a few vascular shadows in the mass interior, indistinct from the left pararenal fascia. The mass measured ~32 × 24 × 7 cm, causing the compression and pushing of the left kidney, pancreas, spleen, and intestinal canals in the abdominal cavity, especially the left half of the colon (Fig. 1A and B). Based on the CT results, it is considered that the possibility of angiomyolipoma is high. Percutaneous biopsy of the lesion was not performed due to limitations of sampling and possible implant metastasis caused by the puncture. An open surgery was performed to excise the lesion. During the open surgery, it was discovered that the tumor was connected to the fat capsule at the lower pole of the left kidney and had grown into the abdominal cavity by breaking through the left renal fascia. No tumor invasion was observed in the renal parenchyma macroscopically. Therefore, the tumor was completely removed along with the cleaning of the perirenal fat capsule (Fig. 1C), and the left kidney was preserved. The cut surface of the tumor was yellow-white, hard in texture (Fig. 1D). Postoperative pathology (Fig. 2) confirmed well-differentiated liposarcoma and negative margins. Immunohistochemistry indicated positivity for MDM2, CDK4, P16, and S-100, with a low proliferation index (Ki-67 ~ 2%). The patient recovered well after the operation. The symptoms of constipation gradually disappeared. The abdominal CT showed that no recurrence was observed during the 12-month follow-up after the operation (Fig. 3).

## Discussion

Perirenal liposarcoma is often asymptomatic in the early stages due to its anatomical location. As the tumor progressively enlarges, patients may develop compressive symptoms affecting various organs (e.g. gastrointestinal obstruction, urinary tract obstruction, lower back and abdominal pain). Most patients only seek medical attention when clinical symptoms manifest. Even with aggressive surgical resection upon diagnosis, the recurrence rate remains high, necessitating prolonged postoperative follow-up. Current surgical consensus mandates complete resection of perirenal liposarcoma, yet the optimal extent of surgical resection remains controversial. In a study involving 228 patients with RLPS undergoing surgery, those requiring multiorgan resection exhibited a lower 10-year survival rate of 26% compared to patients without the need for multi-organ resection 35% [7]. Disregarding tumor size and patient tolerance, multiorgan resection is often associated with higher complication and mortality rates [8]. Therefore, we intraoperatively opted for kidney preservation and complete resection of the perirenal liposarcoma in this case, with postoperative pathology confirming an R0 resection.

## Conclusion

Perirenal liposarcoma is a malignant tumor with a high recurrence rate. Even if the tumor is completely removed, the 5-year survival rate is still poor. Therefore, regular (3–6 months/once) and long-term (at least 5 years) follow-up management must be carried out after surgery. The damage control surgery that aims to completely remove the tumor, preserve organ functions, reduce surgical trauma, and minimize the complications and mortality during the perioperative period should be given priority consideration. In this case, the postoperative time was short, and we will follow up closely to provide more reference for clinical practice.
